# Self-healing juvenile cutaneous mucinosis: A self-resolving dermatologic disorder

**DOI:** 10.1016/j.jdcr.2025.07.024

**Published:** 2025-08-22

**Authors:** Nancy Shehata, Sultana Abdulghani, Husna Irfan Thalib, Areen Murshid

**Affiliations:** a Department of Dermatology, King Abdullah Medical Complex, Jeddah, Saudi Arabia; b General Medicine Practice Program, Batterjee Medical College, Jeddah, Saudi Arabia; c Department of Pathology, King Abdullah Medical Complex, Jeddah, Saudi Arabia

**Keywords:** cutaneous, juvenile, mucinosis, self-healing

## Case description

**Fig 1:**
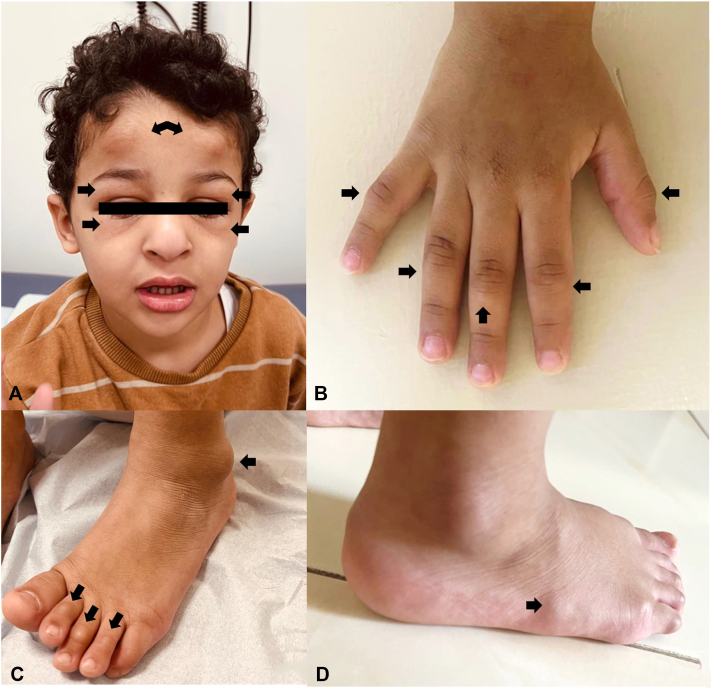
**A,** Frontal nodular lesions with associated eyelid swelling. **B,** Nodular lesions on the interphalangeal joints of the hands. **C** and **D,** Nodular lesions on the interphalangeal joints of the feet, with nodular involvement on the lateral side of the right foot.

**Fig 2:**
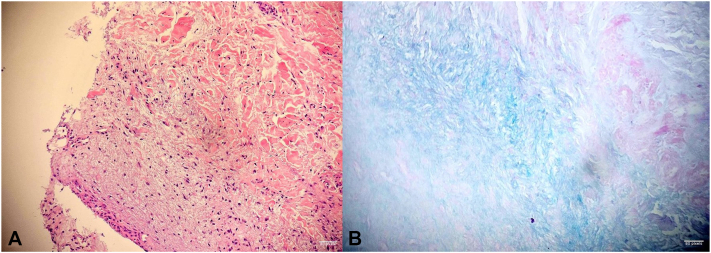
**A,** Histological section of acral skin showing hyperorthokeratosis, hypergranulosis, acanthosis, and focal papillomatosis, with notable dermal spacing due to increased interstitial mucin deposition. **B,** Alcian blue/periodic acid-Schiff stain highlighting mucin accumulation. No evidence of granulomas, parasitic organisms, eosinophilic deposits, dysplasia, or malignancy.

**Fig 3:**
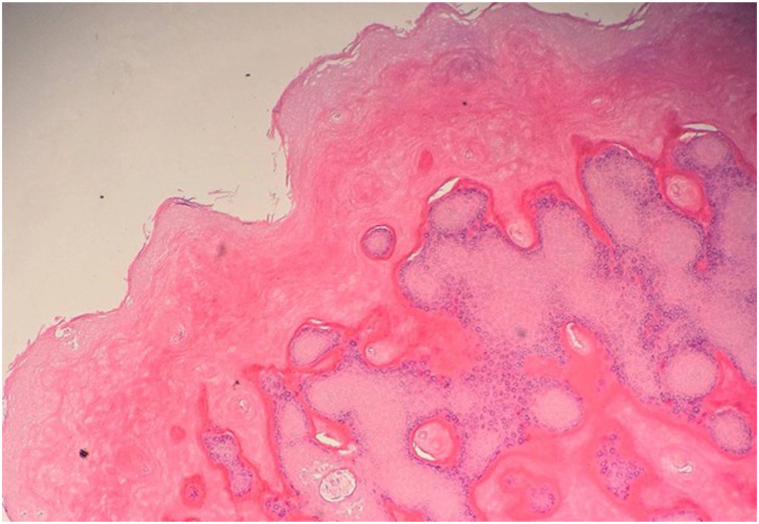
The epidermis shows hyperorthokeratosis, hypergranulosis, acanthosis, and focal papillomatosis (hematoxylin and eosin, ×10). The dermis reveals perivascular infiltration of lymphocytes and a few eosinophils.

**Fig 4:**
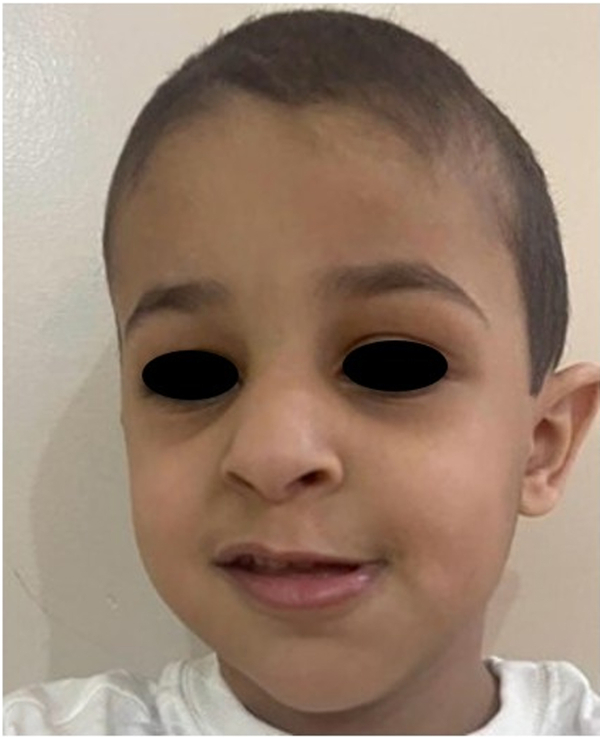
Postresolution image showing complete disappearance of nodules without scarring or pigmentation.

A 4-year-old boy presents with a 1-month history of firm, movable, nontender subcutaneous nodules on the face and extremities, gradually increasing in number, along with intermittent periorbital edema (Fig 1). He sequentially developed subcutaneous nodules, first on his forehead, followed by involvement of his hands and feet, over the course of a few weeks. He has no systemic symptoms. A skin biopsy was taken from a deep lesion on the right foot, which reveals abundant dermal mucin deposition with Alcian blue positivity (Fig 2). The epidermis shows hyperorthokeratosis, hypergranulosis, acanthosis, and focal papillomatosis (hematoxylin and eosin, ×10). The dermis reveals perivascular infiltration of lymphocytes and a few eosinophils (Fig 3). The eosinophil count was 0.24 × 10^9^/L, within normal limits. The case has a history of consanguinity. The nodules resolved within 4-6 weeks without scarring (Fig 4).

**What is the most likely diagnosis?**

**A.** Juvenile sarcoidosis
**B.** Scleromyxedema
**C.** Self-healing juvenile cutaneous mucinosis
**D.** Farber disease
**E.** Non-Langerhans cell histiocytosis

## Discussion

The correct answer is C. Self-healing juvenile cutaneous mucinosis (SHJCM), a rare, benign condition first described in 1973. SHJCM's hallmark features include (1) sudden eruption of firm, nontender subcutaneous nodules preferentially involving periarticular areas and face, (2) characteristic periorbital edema, which is typically intermittent and transient, coinciding with the acute phase of mucin accumulation, and (3) spontaneous resolution within months without scarring. Histopathology is diagnostic, demonstrating mucin deposition in the reticular dermis with Alcian blue positivity at pH 2.5, accompanied by a mild perivascular lymphocytic infiltrate and increased fibroblasts.^1^, ^2^, ^3^ Unlike juvenile dermatomyositis (A) or systemic lupus erythematosus (B), SHJCM lacks myositis, photosensitivity, or autoantibodies.

The pathophysiology remains unclear, but proposed mechanisms include transient fibroblast dysfunction leading to excessive hyaluronic acid production. While about 30% of cases show elevated eosinophils, this finding is nonspecific. In our patient, eosinophil levels were normal.

Key differentials include Farber disease (D), a rare autosomal recessive lysosomal storage disorder caused by *ASAH1* mutations leading to acid ceramidase deficiency. It typically presents in infancy with painful and progressive joint contractures, subcutaneous lipogranulomas, and a hoarse voice due to laryngeal involvement. Histologically, it is marked by granulomatous inflammation, which is distinct from the mucin-rich pattern seen in SHJCM.^1^^,^^4^^,^^5^ Non-Langerhans cell histiocytosis (E) represents a group of disorders such as juvenile xanthogranuloma or Rosai-Dorfman disease, which can present with cutaneous or systemic nodules but are histologically characterized by histiocytic infiltrates rather than mucin deposition, helping to differentiate them from SHJCM.^6^

Although consanguinity was noted in our patient, SHJCM appears to be a sporadic condition. No definitive pattern of familial inheritance or pathogenic mutations has been identified in the literature, confirming that most cases occur without a familial predisposition.^1^^,^^2^^,^^7^, ^8^, ^9^ Clinically, SHJCM is significant for its potential misdiagnosis as autoimmune or metabolic disorders. Treatment is generally unnecessary beyond reassurance, though antihistamines may offer transient relief from pruritus in some cases.^8^

While SHJCM typically resolves spontaneously within months, recent reports suggest possible delayed rheumatologic manifestations. The largest case series by Luchsinger et al, involving nine patients, documented two who later developed fibroblastic rheumatism and an autoinflammatory condition.^1^ Similarly, a 2023 report by Rodríguez-Cuadrado et al described divergent outcomes: 1 patient had full resolution without complications, while another developed idiopathic juvenile arthritis postresolution.^9^ These observations underscore the importance of long-term follow-up to monitor for systemic or rheumatologic disease evolution.^2^ Bolognia's Dermatology (5th ed., p. 758) also advises continued monitoring due to the risk of delayed manifestations.^10^

SHJCM must be carefully differentiated from papulonodular mucinosis associated with systemic lupus erythematosus, which may appear similar but typically features positive autoantibodies and systemic involvement. Histopathologic analysis is critical, particularly to rule out granulomas, dysplasia, or plasma cell-rich infiltrates, preventing misdiagnosis and inappropriate immunosuppression.

In summary, although SHJCM usually resolves without treatment, reports of delayed systemic involvement necessitate careful diagnosis and ongoing clinical follow-up. This case highlights the diagnostic value of histopathology in pediatric dermatology, reinforcing the need for accurate clinicopathologic correlation to prevent mismanagement and ensure appropriate care.

## Conflicts of interest

None disclosed.
